# Therapeutic Potential of Mesenchymal Stem Cells in a Pre-Clinical Model of Diabetic Kidney Disease and Obesity

**DOI:** 10.3390/ijms22041546

**Published:** 2021-02-04

**Authors:** Christian Sávio-Silva, Poliana E. Soinski-Sousa, Antônio Simplício-Filho, Rosana M. C. Bastos, Stephany Beyerstedt, Érika Bevilaqua Rangel

**Affiliations:** 1 Hospital Israelita Albert Einstein, São Paulo 05652-900, Brazil; christian.silvio@einstein.br (C.S.-S.); poliana.soinsk@einstein.br (P.E.S.-S.); antonio.simplicio@einstein.br (A.S.-F.); rosana_cardoso@yahoo.com (R.M.C.B.); stephany.beyerstedt@einstein.br (S.B.); 2 Nephrology Division, Universidade Federal de São Paulo-Escola Paulista de Medicina, São Paulo 04023-900, Brazil

**Keywords:** mesenchymal stem cells, diabetic kidney disease, mitochondria, oxidative stress

## Abstract

Diabetic kidney disease (DKD) is a worldwide microvascular complication of type 2 diabetes mellitus (T2DM). From several pathological mechanisms involved in T2DM-DKD, we focused on mitochondria damage induced by hyperglycemia-driven reactive species oxygen (ROS) accumulation and verified whether mesenchymal stem cells (MSCs) anti-oxidative, anti-apoptotic, autophagy modulation, and pro-mitochondria homeostasis therapeutic potential curtailed T2DM-DKD progression. For that purpose, we grew immortalized glomerular mesangial cells (GMCs) in hyper glucose media containing hydrogen peroxide. MSCs prevented these cells from apoptosis-induced cell death, ROS accumulation, and mitochondria membrane potential impairment. Additionally, MSCs recovered GMCs’ biogenesis and mitophagy-related gene expression that were downregulated by stress media. In BTBR*^ob/ob^* mice, a robust model of T2DM-DKD and obesity, MSC therapy (1 × 10^6^ cells, two doses 4-weeks apart, intra-peritoneal route) led to functional and structural kidney improvement in a time-dependent manner. Therefore, MSC-treated animals exhibited lower levels of urinary albumin-to-creatinine ratio, less mesangial expansion, higher number of podocytes, up-regulation of mitochondria-related survival genes, a decrease in autophagy hyper-activation, and a potential decrease in cleaved-caspase 3 expression. Collectively, these novel findings have important implications for the advancement of cell therapy and provide insights into cellular and molecular mechanisms of MSC-based therapy in T2DM-DKD setting.

## 1. Introduction

Diabetes mellitus (DM) is a globally pandemic metabolic syndrome (prevalence of 9.3%, corresponding to 463 million adults aged 20–79 years, in 2019), with increasing annual worldwide prevalence at 10.9% (700 million) by 2045 [1]. Highly associated with metabolic diseases and a major risk factor for type 2 DM (T2DM), obesity has also reached pandemic levels in the last decades and contributes to aggravating the overall scenario [2]. T2DM (90% of all cases of DM) is a multifactorial disease, mainly characterized by hyperglycemia and insulin resistance, and ultimately to insulin secretion impairment. The well-established gold standard protocol to treat people affected by T2DM is strict glycemic control associated with lifestyle changes, as a risk reduction strategy [3]. However, uncontrolled and persistent hyperglycemia overwhelms metabolic pathways, leading to mesangial cell hypertrophy and proliferation, glomerular basement membrane thickening, podocytopathy, endothelial dysfunction, chronic inflammation, autophagy dysregulation, fibrosis, and oxidative stress signaling activation, triggering pathophysiological complications involved in diabetic kidney disease (DKD) progression [3,4].

DKD, one of the main DM microvascular complications, affects 30–40% of people with DM independently of treatment [4]. Besides increasing cardiovascular disease risk, DKD is the main cause of end stage kidney disease (ESKD) despite any conventional therapy being applied, which means time-dependent loss of kidney function and obligated substitutive intervention, including dialysis or kidney transplantation [5]. Essentially, DKD is a chronic and progressive condition, characterized by a progressive decrease in glomerular filtration rate with persistent and elevated proteinuria, which are associated with structural kidney damage [6].

Mitochondria are firstly and especially affected by hyperglycemia-driven reactive oxygen species (ROS) accumulation and redox disturbed signaling, being classified as one of the major factors leading to DM pathophysiology hallmarks [7]. Damaged mitochondria respond to stress by two mechanisms: a) molecular; synthesizing antioxidant enzymes and b) morphological; orchestrating the processes of biogenesis, mitophagy, fusion, fission, and motility [8]. Persistent stress overcomes mitochondria adaptation responses, leading to damage and dysfunction [9]. Dysfunctional mitochondria itself causes excessive ROS production, which affects remaining healthy mitochondria segments and possibly triggers a feedback-based vicious cycle of increasing damage, eventually leading to cell energy collapse [10]. As an alternative to clean up the dysfunctional mitochondria fragments and avoid energetic fail, mitophagy is a key player in preventing cell collapse and death induction, possibly as an important therapeutic target [11].

In order to upgrade DM-DKD treatment, mesenchymal stem cells (MSCs) have great reparative potential as a complement therapy to gold standard protocol [12]. Substantially, MSCs have demonstrated anti-oxidative and anti-apoptotic effects [13], through their secretome and mitochondria transfer to neighboring in-contact damaged cells [12]. MSCs promote anti-oxidant enzymes expression, decrease ROS production, and contribute to mitochondrial repair [14,15] and cell death inhibition [16]. In that way, we aimed to apply MSCs in a T2DM-DKD and obesity pre-clinical model using leptin-deficient BTBR*^ob/ob^* (black and tan, brachyuric) mice, and verify their therapeutic potential for inhibiting oxidative stress and cell death over induction, as well as the contribution towards halting DKD progression.

## 2. Results

### 2.1. Mesenchymal Stem Cell (MSC) Characterization

**Figure 1:**
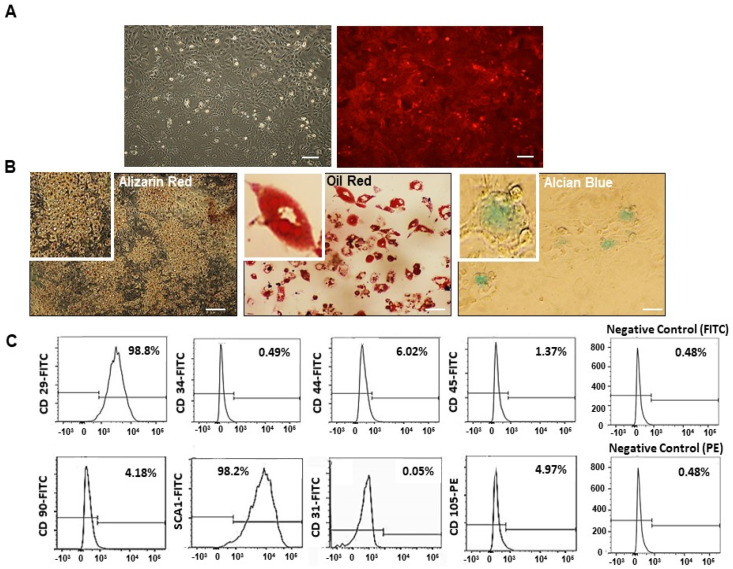
Characterization of bone marrow-derived mesenchymal stem cells (MSC) isolated from mTmG mice. (**A**) Plastic adherent cells were ex vivo expanded until passage 10, maintaining red fluorescent (RFP) expression throughout. (**B**) MSCs differentiated into osteogenic (alizarin red staining), adipogenic (oil red staining), and chondrogenic (alcian blue staining) lineages. Scale bars represent 200 µm. (**C**) Flow cytometry analysis for immunophenotyping showed that MSCs, in passages 7–8, were positive for CD 29 (98.8%), SCA-1 (98.2%), CD 44 (6.02%), CD 90 (4.18%), and CD 105 (4.97%), and negative for CD34 (0.49%), CD 45 (1.37%), and CD 31 (0.05%).

We have successfully isolated and purified MSCs obtained from bone marrow (BM) of male mTmG mice. We characterized these cells through analysis of RFP^+^ expression maintenance on cell membranes, according to serial cell culture passages (Figure 1A); capacity of multi-differentiation capacity into osteogenic, adipogenic, and chondrogenic lineages (Figure 1B); and immunophenotyping analysis, confirming the expression of stem cell antigen markers and negative expression of hematopoietic lineage markers (Figure 1C).

### 2.2. Mitochondrial Accumulation of H_2_O_2_ in Glomerular Mesangial Cells (GMCs) Following Stress Media Conditioning

**Figure 2:**
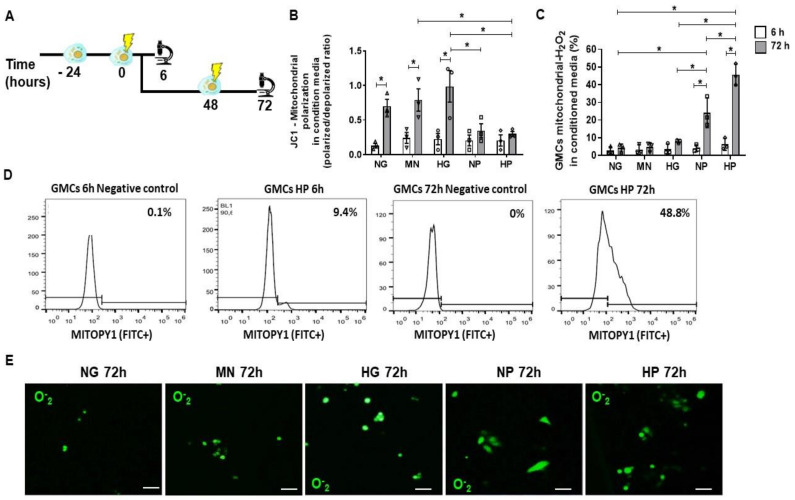
Oxidative stress in GMCs at mitochondrial level after stress-conditioning. (**A**) Scheme of the cellular experiments for GMC conditioning with stress-conditioning media at different time-points (6 h and 72 h). (**B**) Flow cytometry analysis for mitochondria polarization ratio in stress-conditioned glomerular mesangial cells (GMCs) after different time points (6 h and 72 h; polarized/depolarized ratio). Hyper glucose (HG) caused hyperpolarization of GMCs mitochondria in comparison with normal glucose (NP) and hyper glucose + hydrogen peroxide (HP) conditioning, which caused a depolarization in comparison to control media (* *p* < 0.05). (**C**) Flow cytometry analysis of hydrogen peroxide (H_2_O_2_) accumulated specifically into GMC-mitochondria (FITC^+^) after stress-conditioning media. NP and HP 72 h-conditioning caused expressive H_2_O_2_ accumulation in GMCs mitochondria (* *p* < 0.05). Groups were compared with each other intra and inter each time-point (6 h and 72 h). (**D**) Flow cytometry gating and negative control for mitochondrial-H_2_O_2_ accumulation in GMCs conditioned for 6 and 72 h in HP medium. (**E**) Superoxide (O_2_^−^) accumulation in GMCs after 72 h in stress-conditioning media in NP and HP, as demonstrated by confocal microscopy (FITC^+^). Scale bars represent 200 µm. Specific accumulation of H_2_O_2_ in mitochondria (FITC^+^), mitochondria polarization ratio (polarized-RFP+/depolarized-FITC^+^) and superoxide anion cell-accumulation (FITC^+^) in GMCs conditioned to stress media (normal glucose—NG, mannitol—MN, hyper glucose—HG, normal glucose + hydrogen peroxide—NP, hyper glucose + hydrogen peroxide—HP) during 6 h and 72 h time-points. Error bars represent mean ± SEM; *n* = 3. * *p* < 0.05.

We assessed the oxidative stress impact of the conditioning media on GMCs and GMCs’ mitochondria after 6 h and 72 h time-points. In the 6 h time-point, we aimed to evaluate the acute stress, while in the 72 h time-point, when the cells were exposed twice to the stress media, a chronic scenario associated with a repeated insult was mimicked. In Figure 2A, we described the schemes of these cellular experiments. Hyper glucose (HG) media resulted in an inducing effect, causing an increase in the mitochondrial polarization ratio after 72 h treatment, which indicates a possible higher demand of mitochondria working force (*p* < 0.05). On the other hand, NP (normal glucose + hydrogen peroxide) and HP (hyper glucose + hydrogen peroxide) media had a halting effect on mitochondria polarization throughout the 72 h culture evaluation in this mitochondrial function parameter, evidencing a possible damage-dependent loss of function (*p* < 0.05) (Figure 2B). GMCs had severe H_2_O_2_ accumulation into mitochondria after NP and HP 72 h-conditioning, illustrating a significant oxidative stress scenario (*p* < 0.05) (Figure 2C,D). As expected, HP conditioning is the worst scenario when compared to other groups (*p* < 0.05), indicating an additive effect of hyper glucose and H_2_O_2_. Additionally, NP and HP stress-conditioning caused an elevated O_2_^−^ accumulation in GMCs’ cytoplasms in comparison to control groups (Figure 2E). Mannitol was used for osmolarity control (MN), whereas normal glucose (NG) was the control group.

### 2.3. Anti-Oxidative Effect of MSCs Co-Culture on GMCs Conditioned with Stress Media

**Figure 3:**
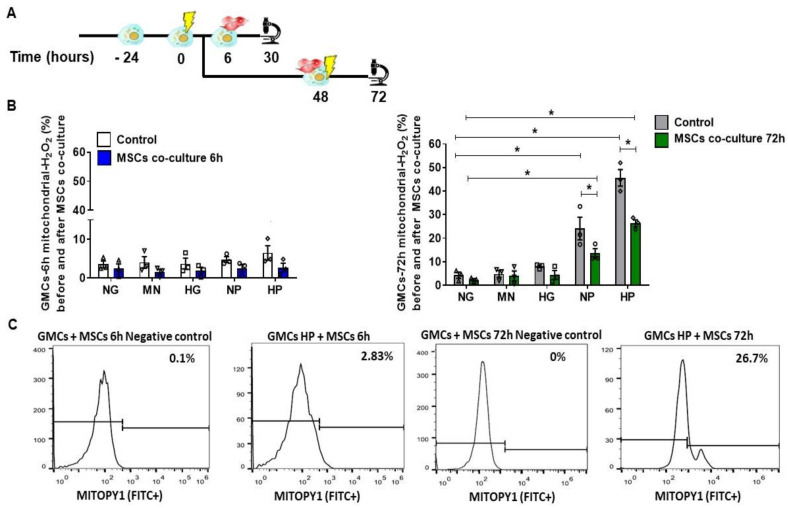
Anti-oxidative effect of MSCs co-culture on GMCs conditioned with stress media. (**A**) Scheme of the cellular experiments for GMCs conditioning with stress-conditioning media at different time-points (6 h and 72 h) and the subsequent MSC co-culture 24 h-therapy. (**B**) Mitochondrial accumulation of H_2_O_2_ after 6 h and 72 h conditioning following MSCs co-culture 24h-therapy. NP and HP media had significantly diminished ROS accumulation in the 72 h time-point (* *p* < 0.05). (**C**) Flow cytometry gating and negative control for mitochondrial-H_2_O_2_ accumulation in GMCs conditioned for 6 h and 72 h in HP medium. Specific accumulation of H_2_O_2_ in mitochondria (FITC^+^) of GMCs conditioned to stress media after MSCs co-culture (normal glucose—NG, mannitol—MN, hyper glucose—HG, normal glucose + hydrogen peroxide—NP, hyper glucose + hydrogen peroxide—HP) during 6 h and 72 h time-points. Error bars represent mean ± SEM; *n* = 3, * *p* < 0.05.

To examine the anti-oxidative potential of MSC treatment on GMCs, we assessed the mitochondrial ROS accumulation after growing these cells with NP and HP media. In Figure 3A, we described the schemes of these cellular experiments with co-culture. Importantly, MSC co-culture, as a 24 h-treatment, was able to inhibit the H_2_O_2_ mitochondrial accumulation after 72 h conditioning, evidencing a notable anti-oxidative capacity (*p* < 0.05) (Figure 3B,C).

### 2.4. GMCs Death Induction after Stress-Conditioning Media

**Figure 4:**
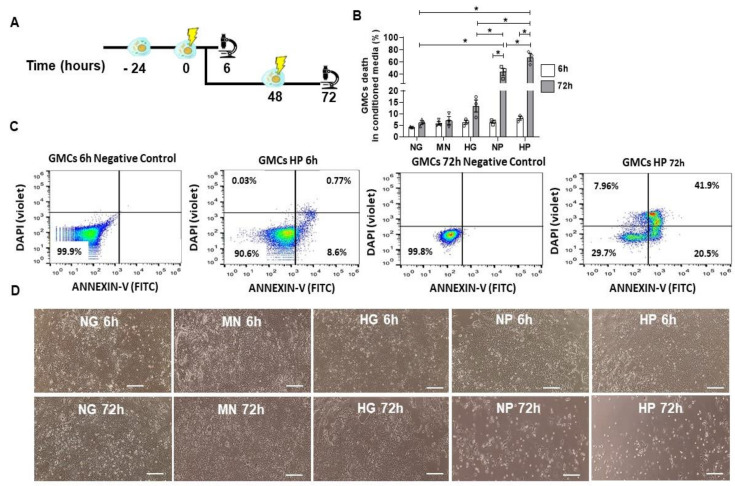
GMCs’ death induction after stress-conditioning at different time points. (**A**) Scheme of GMCs’ experiments for stress-conditioning at different time-points (6 h and 72 h). (**B**) Flow cytometry analysis of GMCs’ death after stress-media conditioning. NP and HP media caused an increase in cell death, in comparison to 6 h conditioning (* *p* < 0.05). (**C**) Flow cytometry gating and negative control for GMCs conditioned to HP media at 6 h and 72 h. (**D**) Light microscopy imaging of GMCs conditioned at 6 h and 72 h. NP and HP caused a visual morphology alteration, disturbing cell growth and culture plate occupancy after 72 h conditioning. Scale bars represent 200 µm. Cell death (Annexin-V^+^ and DAPI^+^) in GMCs conditioned to stress media (normal glucose—NG, mannitol—MN, hyper glucose—HG, normal glucose + hydrogen peroxide—NP, hyper glucose + hydrogen peroxide—HP) during 6 h and 72 h time-points. Error bars represent mean ± SEM; *n* = 3.

Next, we verified the impact of stress-conditioning media on cell death. In Figure 4A, we described the schemes of the cellular experiments. NP and HP markedly induced cell death (*p* < 0.05) in GMCs after 72 h-conditioning (Figure 4B). As expected, HP conditioning is the worst scenario when compared to other groups (*p* < 0.05), indicating an additive effect of hyper glucose and H_2_O_2_. For flow cytometry analysis, we used Annexin-V and DAPI to assess cell death for apoptosis and early necrosis assessment, respectively (Figure 4C). NP and HP media caused severe disturbances in GMCs’ morphology and growth after 72 h-conditioning (Figure 4D), inhibiting plate occupancy and negatively impacting cell shape by reducing cytoplasm and causing loss of fibroblast-like conformation. In the same time-point, HG-conditioned GMCs had similar alterations, but to less extent, such as disturbed growth on culture by cell stress induction and mild cell death increase.

### 2.5. Pro-Survival and Protective Effect of MSCs Co-Culture on Stress-Conditioned GMCs

**Figure 5:**
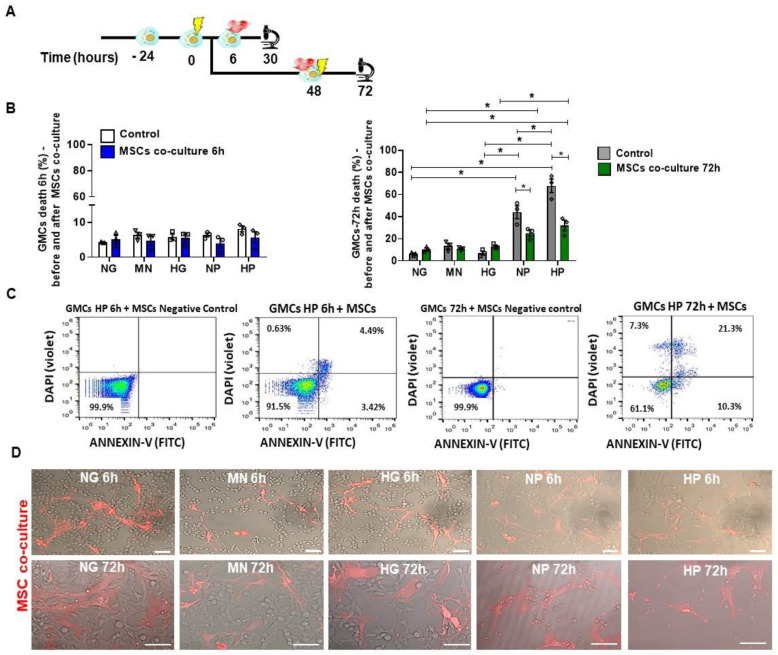
Pro-survival effect of MSCs co-culture on stress-conditioned GMCs. (**A**) Scheme of the cellular experiments for GMC conditioning with stress media-conditioning at different time-points (6 h and 72 h) and the subsequent MSCs co-culture therapy (24 h). (**B**) Flow cytometry analysis of GMC death (%) after MSC co-culture at 6 h and 72 h time-points, respectively. MSCs co-culture diminished GMCs’ death (%) after 72 h stress-conditioning with NP and HP media (* *p* < 0.05). (**C**) Flow cytometry gating and negative control for GMCs conditioned to HP at 6 h and 72 h time-points, with Annexin-V and DAPI staining. (**D**) Confocal images of MSC co-culture with stress media-conditioning at different time-points (6 h and 72 h). Scale bars represent 50 µm. Cell death (FITC^+^ and DAPI^+^) in GMCs conditioned to stress media (normal glucose—NG, mannitol—MN, hyper glucose—HG, normal glucose + hydrogen peroxide—NP, hyper glucose + hydrogen peroxide—HP) during 6 h and 72 h time-points. Error bars represent mean ± SEM; *n* = 3.

To verify the anti-cell death potential of MSC treatment on GMCs, we assessed the apoptosis (Annexin) and early necrosis (DAPI) after growing these cells with NP and HP media. In Figure 5A, we described the schemes of these cellular experiments with co-culture. Importantly, MSC co-culture, as a 72 h-treatment, was able to decrease cell death in NP and HP media (*p* < 0.05) (Figure 5B–D), highlighting notable therapeutic potential of MSCs.

### 2.6. Metabolic and Renal Functional Parameters in BTBR^ob/ob^ Control and MSC-Treated BTBR^ob/ob^ Mice

**Figure 6:**
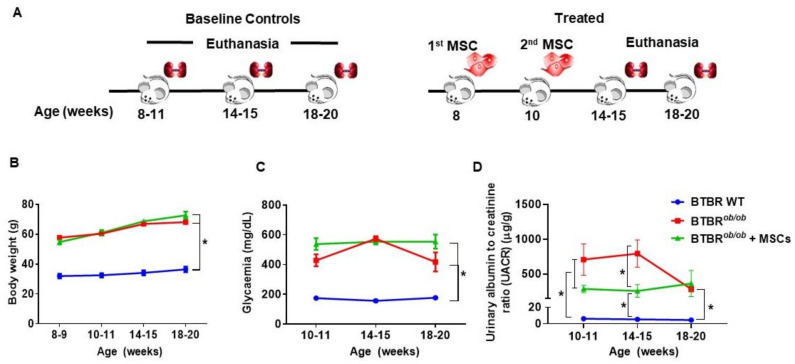
Metabolic and renal functional parameters in BTBR*^ob/ob^* control and MSC-treated BTBR*^ob/ob^* mice. (**A**) Schemes of the procedure. Blood and urine samples were collected at three time-points in accordance to animal age (8–11, 14–15, and 18–20 weeks old) for both BTBR*^ob/ob^* control group and MSC-treated BTBR*^ob/ob^* group. MSCs were injected in the 8th and 10th weeks of age and euthanasia was performed in the 18th–20th weeks of age. (**B**) Body weight gain in BTBR*^ob/ob^* was not prevented by MSC treatment (*p* > 0.05). (**C**) Glycemia after 6 h-starvation did not decrease after MSC treatment (*p* > 0.05). (**D**) Urine-albumin-to-creatinine ratio (UACR) was significantly diminished in MSC-treated BTBR*^ob/ob^* mice in the 14th week of age when compared to BTBR*^ob/ob^* control mice (* *p* < 0.05). Error bars represent mean ± SEM; *n* = 6 animals.

To further substantiate the therapeutic potential of MSC on DKD progression, we injected these cells into BTBR*^ob/ob^* mice at two time-points (8 weeks and 10 weeks). In Figure 6A, we illustrated the experimental protocol throughout the time. There were no evidences of MSCs’ effect in BTBR*^ob/ob^* mice’s natural obesity (Figure 6B) and glucose control (Figure 6C), as an impact on time-dependent body weight gain and hyperglycemia (*p* > 0.05), yet a trend towards lower glycemic levels at 18–20 weeks after MSC treatment was observed. To note, MSCs significantly reduced urine albumin-to-creatinine ratio (UACR) in MSC-treated BTBR*^ob/ob^* mice, showing an important renoprotective effect preventing the elevated albuminuria present in 14 week-old BTBR*^ob/ob^* mice (*p* < 0.05) (Figure 6D).

### 2.7. RNA Expression of Mitochondria Quality Control Program (MQCP)-Related Genes in GMCs and in BTBR^ob/ob^ Mice Kidney Cortexes

**Figure 7:**
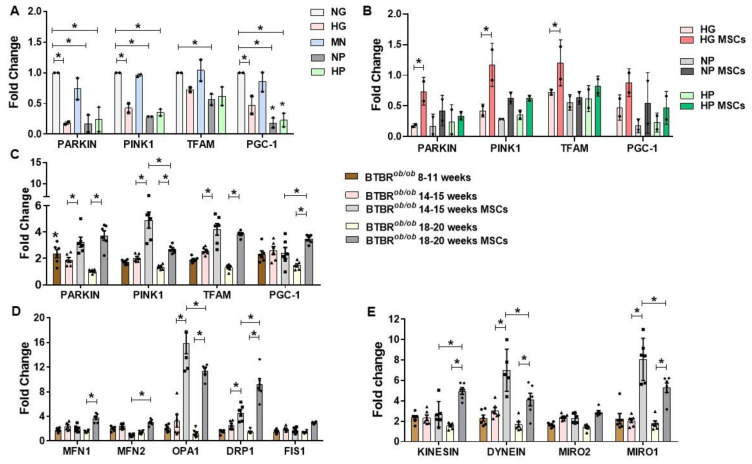
RNA expression of mitochondria quality control program (MQCP)-related genes in GMCs and in BTBR*^ob/ob^* mice’s kidney cortexes. (**A**) GMCs cultured in stress-conditioning media (HG, NP, and HP) presented a down-regulation in PARKIN and PINK1 gene expression at 72 h, whereas PGC-1 expression was only inhibited in NP and HP at 72 h (* *p* < 0.05). (**B**) MSCs treatment recovered PARKIN, PINK1, and TFAM gene expression in GMCs after HG 72 h-conditioning (* *p* < 0.05). (**C**) PARKIN, PINK1, TFAM, and PGC-1 genes had decreased expression in 14–15 and 18–20 week-old BTBR*^ob/ob^* mice kidney cortexes in comparison to 10–11 week-old mice. MSC treatment at 14–15 and 18–20 weeks of age upregulated PARKIN, PINK1, and TFAM (*p* < 0.05). PGC-1 was upregulated only in MSC-treated 18–20 week-old mice (* *p* < 0.05). (**D**) DRP1 and OPA1 had increased gene expression in 14–15 and 18–20 week-old MSC-treated BTBR*^ob/ob^* mice kidney cortexes (*p* < 0.05). MFN1 only had increased gene expression in 18–20 week-old MSC-treated BTBR*^ob/ob^* mice kidney cortexes (*p* < 0.05). (**E**) DYNEIN and MIRO1 had increased gene expression in 14–15 and 18–20 week-old MSC-treated BTBR*^ob/ob^* mice kidney cortexes (* *p* < 0.05). KINESIN had only increased gene expression in 18–20 week-old MSC-treated BTBR*^ob/ob^* mice (* *p* < 0.05). Our qRT PCR results (2^ΔΔCt^) were normalized to BTBR wild-type mice at each related age (10–11, 14–15, and 18–20 week-old). Error bars represent mean ± SEM. For GMCs qRT PCR assays, *n* = 2. For BTBR mice qRT PCR assays, *n* = 6.

**Figure A1:**
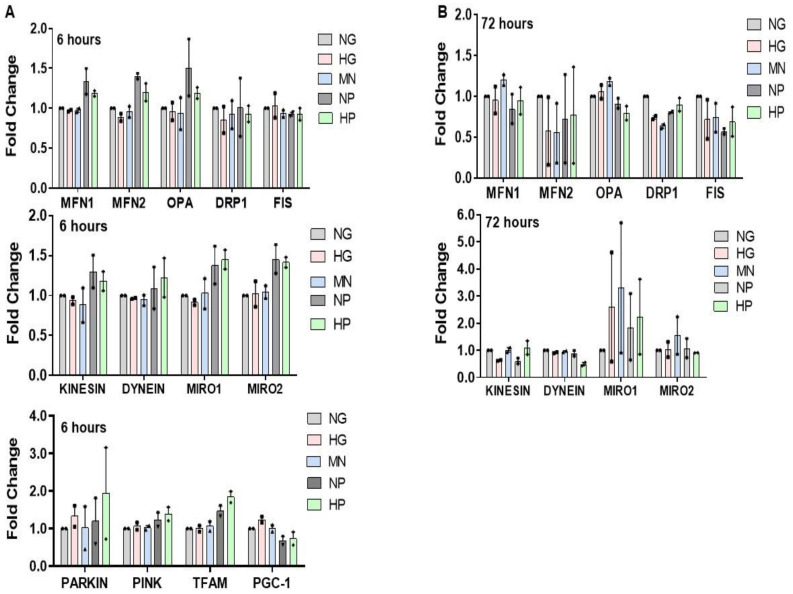
MQCP-genes expression in 6h-GMCs stress-conditioned. (**A**) GMCs stress-conditioned to media during 6h time-point. There was no statistical difference in MQCP-genes expression comparing groups for biogenesis (PGC-1, TFAM), mitophagy (PARKIN, PINK), fusion (MFN1, MFN2, OPA), fission (DRP1, FIS) and motility (KINESIN, DYNEIN, MIRO1, MIRO2) processes (*p* > 0.05; *n* = 2). (**B**) GMCs stress-conditioned to media during 72h time-point. There was no statistical difference in MQCP-genes expression comparing groups for fusion (MFN1, MFN2, OPA), fission (DRP1, FIS) and motility (KINESIN, DYNEIN, MIRO1, MIRO2) processes (*p* > 0.05; *n* = 2).

Next, we hypothesized that MSC might modulate MQCP in DKD setting. Furthermore, 72 h-conditioned GMCs had inhibited relative expression of biogenesis and mitophagy related-genes in HG, NP, and HP media (*p* < 0.05) (Figure 7A). At the earlier time-point (6 h), no differences were noticed in gene expression in accordance to cell culture media (Figure A1A). Additionally, there was no difference in gene expression in 72 h-GMCs processes of MQCP fusion, fission, and motility (Figure A1B). MSC treatment preserved mitophagy-related genes in GMCs conditioned to HG in the 72 h scenario (*p* < 0.05), but not in NP and HP conditioning (Figure 7B).

As DKD progressed in mice, there was a lower expression of MQCP-related gene expression for almost every marker evaluated in older BTBR*^ob/ob^* mice (14 and 20 weeks old) compared to younger BTBR*^ob/ob^* mice (10–11 weeks old) (*p* < 0.05) (Figure 7C–E). MSC-treated BTBR*^ob/ob^* mice (14–15 and 18–20 weeks old) had notably increased change in expression of almost every MQCP-related gene evaluated, indicating a stimulating effect of MSCs therapy on the mitochondrial molecular pathways in mice kidneys and a possible MQCP restoration in DKD scenario (*p* < 0.05) (Figure 7C–E).

### 2.8. MSCs Preserve BTBR^ob/ob^ Kidneys from Mesangial Expansion and Podocyte Loss

**Figure 8:**
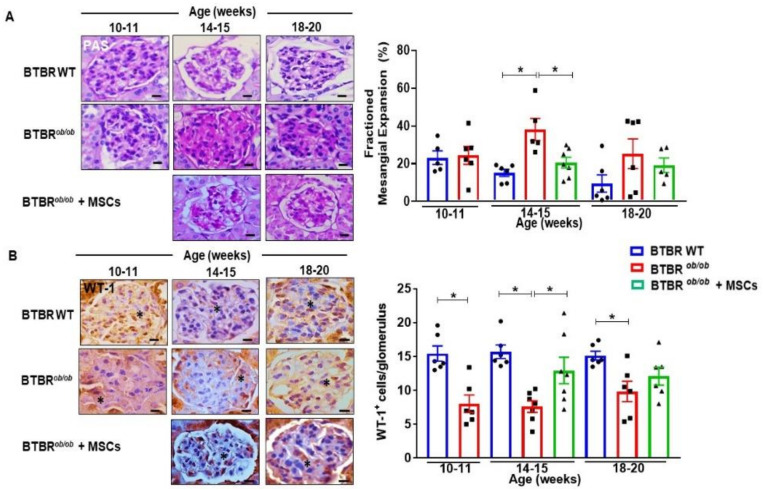
MSCs preserve BTBR*^ob/ob^* kidneys from mesangial expansion and podocyte loss. (**A**) DKD promoted increased matrix deposition in mesangial compartment of BTBR*^ob/ob^* mice at 14–15 weeks of age (* *p* < 0.05). MSC treatment curtailed mesangial expansion when 14–15 week-old MSC-treated BTBR*^ob/ob^* mice were compared to 14–15 week-old BTBR*^ob/ob^* mice (* *p* < 0.05). (**B**) Number of podocytes per glomerulus, verified by the immunohistochemistry staining for WT-1 protein, decreased as DKD progressed in BTBR*^ob/ob^* mice when compared to BTBR WT (*p* < 0.05). MSC treatment showed a protective effect as podocyte maintenance leading to a higher number of WT-1^+^ cells in 14–15 week old MSC-treated BTBR*^ob/ob^* mice in comparison to 14–15 week old BTBR*^ob/ob^* mice (* *p* < 0.05). BTBR WT was not statistically different at 18–20 weeks. (*) indicates WT1^+^ cell. Scale bars represent 10 µm in (A and B). Error bars represent mean ± SEM. For periodic acid-Schiff (PAS) and WT-1 staining: *n* = 5–7 animals.

We further looked into the therapeutic potential of MSC in BTBR*^ob/ob^* mice. Therefore, MSC-treated mice had a significantly lower mesangial matrix deposition at 14–15 weeks of age, reaching 14–15 week-old BTBR (wild-type) WT levels (*p* < 0.05) (Figure 8A). This protective effect was not sustained until 18–20 weeks of age, indicating a time-dependent effect of MSC therapy. A similar outcome was obtained with podocyte/glomeruli maintenance. MSCs preserved podocyte cell loss of BTBR*^ob/ob^* mice at 14–15 weeks of age (*p* < 0.05). This effect was not sustained until 18–20 weeks of age. BTBR*^ob/ob^* mice had lower podocyte/glomeruli numbers in comparison to BTBR WT at every age investigated (*p* < 0.05), demonstrating the hallmark of podocyte loss during DKD progression (Figure 8B). Importantly, the findings of curtailed mesangial expansion and preservation of podocyte numbers in BTBR*^ob/ob^* mice have important therapeutic implications and may explain the lower values of UACR, as previously documented.

### 2.9. MSCs Potentially Suppress Caspase-3 Dependent Cell Death over Induction in BTBR^ob/ob^ Mice Kidney

**Figure 9:**
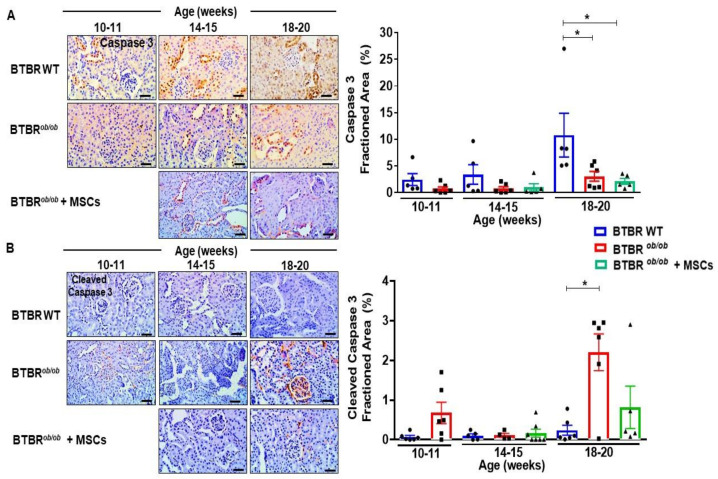
MSCs potentially suppress caspase-3 dependent cell death over induction in BTBR*^ob/ob^* mice kidney. (**A**) Caspase 3 protein was induced in 20-week old BTBR (wild-type) WT mice kidney in comparison to 14-week old BTBR*^ob/ob^* (* *p* < 0.05). (**B**) Cleaved-caspase 3 overexpressed in 20-week old BTBR*^ob/ob^* in comparison to every other group (* *p* < 0.05). Error bars represent mean ± SEM. For caspase-3 and cleaved caspase-3 staining: *n* = 5–6 animals. Scale bars represent 20 µm (**A**,**B**).

To test whether the beneficial outcome of MSCs was also attributed to a decrease in apoptotic cell death, we verified the caspase 3 signaling pathway in kidney cortex sections of BTBR*^ob/ob^* and BTBR wild type mice. Despite caspase 3 induction in 20 week-old BTBR WT mice, when compared to BTBR*^ob/ob^* (*p* < 0.05) (Figure 9A), active cleaved-caspase 3 was up-regulated as DKD progressed in BTBR*^ob/ob^* mice (Figure 9B), indicating activation of this cell death signaling pathway. Therefore, MSC therapy led to a potential decrease in cleaved-caspase 3 expression in 20 week-old BTBR*^ob/ob^* mice (Figure 9B).

### 2.10. MSCs Therapy Reduces Renal LC3 over Expression and Potentially Diminishes Renal Lipid Peroxidation during DKD Progression

**Figure 10:**
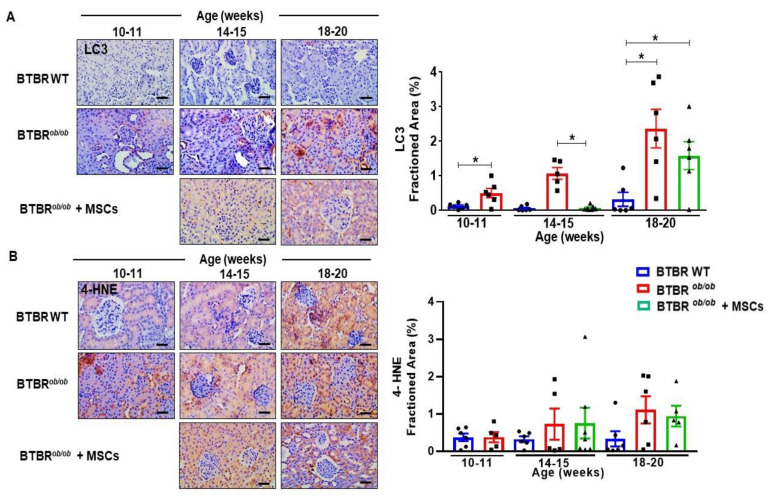
MSC therapy reduces renal LC3 over expression and potentially diminishes renal lipid peroxidation during DKD progression. (**A**) Renal LC3 expression was increased in BTBR*^ob/ob^* at any age compared to BTBR (wild-type) WT mice (* *p* < 0.05). MSC therapy was effective reducing LC3 over induction in 14–15 week-old BTBR*^ob/ob^* mice (* *p* < 0.05). However, the effect did not persist until 18–20 weeks of age. (**B**) 4-HNE staining showed potential increased lipid peroxidation in 14–15 and 18–20 week-old BTBR*^ob/ob^* mice, when compared to BTBR WT. However, MSCs therapy did not reduce oxidative stress in these animals. Error bars represent mean ± SEM. For LC3 and 4-HNE staining: *n* = 5–7 animals. Scale bars represent 20 µm (A and B).

Next, we investigated autophagy signaling pathways during DKD progression. Autophagy marker microtubule-associated protein light chain 3 (LC3) is used to assess autophagy activity and has two isoforms. LC3-I is converted to LC3-II and then to autophagic vesicle. To note, BTBR*^ob/ob^* mice had increased LC3 protein expression in kidney sections by 20 weeks of age (*p* < 0.05), indicating that autophagy increased over time (Figure 10A). We pursued further investigation of LC3 expression in MSC-treated BTBR*^ob/ob^* mice and found lower LC3 expression values in 14–15 week-old treated mice (*p* < 0.05), pointing out a temporary protective effect on suppressing the superlative time-dependent autophagy induction in consequence of DKD progression in these animals, as this effect did not persist until 18–20 weeks of age (Figure 10A). BTBR*^ob/ob^* mice had higher LC3 expression in comparison to BTBR WT mice at every age evaluated (*p* < 0.05).

Finally, we investigated the 4-HNE lipid peroxidation marker as a consequential effect of HG-driven oxidative stress in BTBR*^ob/ob^* kidney. Lipid peroxidation was potentially increased in 14–15 and 1–20 week-old BTBR*^ob/ob^* mice in comparison to 14–15 and 18–20 week-old BTBR WT mice. However, MSC therapy did not curtail oxidative stress in 14–15 and 18–20 week-old BTBR*^ob/ob^* mice with DKD (Figure 10B).

## 3. Discussion

To our knowledge, this is the first study applying MSCs therapy to BTBR*^ob/ob^* mice, which is the most robust T2DM and DKD pre-clinical model [17]. The present study sought to provide direct evidence of MSCs as a therapeutic intervention to halt the progression of DKD. Our main findings were the anti-apoptotic, autophagy modulation, and anti-oxidative effects of MSCs on GMCs, associated with the upregulation of genes related to mitochondrial biogenesis and mitophagy. In vivo, MSC-treated BTBR*^ob/ob^* mice were prevented from elevated albuminuria, had diminished mesangial expansion, and had preserved numbers of podocytes. Additionally, MSCs upregulated MQCP related genes in treated BTBR*^ob/ob^* mice and preserved kidney from LC3-mediated autophagy overexpression.

Currently, DKD natural history has been changed by advances in pharmacological tools, especially due to sodium-glucose cotransporter-2 (SGLT2) inhibition by gliflozins addition to the standard treatment [18]. Specifically, gliflozins preserve kidney function through hemodynamic reestablishment, preventing hyperfiltration and restoration of tubule-glomerular feedback [19]. However, the association of pharmacological interventions with improvements in the life style (diet, exercise, weight control, and quitting smoking) is able to slow disease progression, yet not recovering either lost tissue, function, or both [18]. That is the fundamental gap which MSCs treatment is able to fulfill, presenting a cumulative body of evidences showing safe and effective renoprotective applications [12,20], even though there is methodological variability in research studies, which makes data comprehension about the underlying mechanisms difficult [21].

We successfully applied allogeneic BM-MSC therapy to GMC culture and BTBR*^ob/ob^* mice, a robust and severe T2DM and DKD animal model that mimics key features of advanced human DKD. We have properly extracted, purified, characterized, and safely administered MSCs to both in vitro and in vivo experimental design. In our study, there was a damaging effect of the conditioning associated with the chronicity (6–72 h) of stress-media (HG, NP, and HP) on GMCs. Specifically, HP medium exhibited the most negative impacts, showing an additive association of HG and H_2_O_2_ action in comparison to HG and NP media. Increased glucose influx (HG) leads to higher NADH and FADH2 synthesis and persistently increases mitochondrial membrane potential and causes disturbance to the electron transport chain (ETC), consequently increasing electron leakage that culminates in higher superoxide (O_2_^−^) and H_2_O_2_ radicals production [22,23]. That oxidative scenario due to DM-induced metabolite changes explains our findings regarding GMCs’ mitochondrial membrane alterations in association with O_2_^−^-H_2_O_2_ accumulation and cell death induction. Accumulated O_2_^−^-H_2_O_2_ causes mitochondrial disturbance through redox balance alterations in ETC, culminating in energy failure and inducing cell death [24]. Physiologically, cells sustain a stable mitochondrial membrane potential, with a tight control mechanism of ROS concentration [23], yet transient alterations are common and not injurious during adaptation to environmental changes [25]. Sustained H_2_O_2_ and O_2_^−^ accumulation is deleterious to the mitochondrial homeostasis, overcoming stress response and adaptation processes like mitophagy, eventually leading to cell death [9]. Correspondingly, we evidenced a diminished fold change expression of genes related to both mitophagy and biogenesis in GMCs facing persistent HG, NP, and HP conditioning. On the other hand, MSC-treated GMCs were successfully preserved from cell death over induction by stress media conditioning, especially and more notably in relation to the prolonged and accumulated effect of the HP medium. On the other hand, MSCs have been well described as effective anti-apoptosis and anti-necrosis therapeutic tools in oxidative stress and hyperglycemic scenarios of DKD [26,27]. According to our results, MSCs are reported to respond more effectively towards repair when challenged or primed in a stress condition, triggering mechanisms that culminate in a more reparative secretome composition [28]. Additionally, injured cells release mitochondria or mitochondria fragments into the extracellular microenvironment as a damage signal, these mitochondria are then engulfed by neighboring MSCs. Internalized exogenous mitochondria elicit MSCs’ anti-apoptotic response in association with increased biogenesis and mitochondria donation to the damaged cells, culminating in an anti-oxidative effect as well [29]. Likewise, we have also found MSCs efficiently inhibiting H_2_O_2_ accumulation in GMCs, preventing them from oxidative damage and mitochondria dysfunction. Complementing that point, our results showed that MSC-treated GMCs recovered the expression of genes related to biogenesis and mitophagy processes. Importantly, MSCs have reported renoprotective effects through biogenesis enhancing in damaged tubular epithelial cells [30].

Regarding BTBR*^ob/ob^* mice, we decided to adopt fresh thawed cells administration, intraperitoneal route, and allogeneic transplantation as a way to simulate a more susceptible method to be translated for clinical application. As reviewed elsewhere, the MSC administration route is an essential factor impacting the reparative effects [31]. Regularly, MSCs are administered through the intravenous route, which produces a significant renoprotective effect by reducing serum creatinine, though being less effective in comparison to the arterial delivery route [31]. Besides, intravenous injection usually leads to MSCs being trapped in the pulmonary microvasculature, reducing the homing to damaged organs [32]. Intraperitoneal administration is a safe and effective method to apply MSCs therapy, already demonstrated by renoprotective results facing kidney injury, with anti-apoptotic, anti-inflammatory [33], and anti-oxidative effects associated with better renal function outcome [34]. The intraperitoneal route allows infused MSCs to migrate and get systemically distributed, including kidney homing in on renal damage scenarios [33]. We obtained promising results of MSC therapy effects in the T2DM-DKD pre-clinical model using BTBR*^ob/ob^* mice. We observed an important time-dependent effect relative to T2DM and DKD injury. Nevertheless, we evidenced a protective effect of MSC therapy on BTBR*^ob/ob^* mice. First and most relevant, MSC therapy was renoprotective, suppressing the elevated albuminuria naturally seen in DKD progression. Secretome from MSCs can drive pro-survival cascade events, inhibiting cell damage through repair pathways and avoiding cell death induction [35]. Additionally, damaged kidney signaling, through chemokines and stress molecules, exerts an attraction on MSCs homing and drives them directly to the renal region, promoting a temporary engraftment [36]. The mentioned engraftment allows direct MSC-kidney cells contact and communication, favoring mitochondria transferring from one to another [37]. MSCs mitochondria transfer is one of the main repair mechanisms that underlies the anti-oxidative and anti-apoptotic effects [37,38]. Evidences confirm the renoprotective effect of MSCs on DKD, demonstrated as kidney preservation in pre-clinical T1DM and T2DM models after systemic administration of MSCs or MSC-conditioned media, reflecting in lower proteinuria rate [26]. On the other hand, MSC administration could not change the body weight gain and hyperglycemic profile of BTBR*^ob/ob^* mice, yet there was a trend towards lower levels of glycemia in BTBR*^ob/ob^* mice at 18–20 weeks. It is important to note that we tested MSCs therapy administered systemically and without any complementary intervention against the severe hyperglycemic and obesity condition of our animal model, what brings great challenges to be overcome but also highlights the obtained effects. Increasing the frequency of MSCs administration over time or an earlier intervention would possibly cause a more prominent and sustained response. Nonetheless, there are compelling evidences of hypoglycemic MSC-therapy effect in T1DM and T2DM pre-clinical models [39,40]. Certainly, an association of MSCs with a gold standard pharmaceutical therapy, such as SGLT2 inhibitors, would bring major upgrades to these effects on hyperglycemia and body weight, besides the expected greater kidney improvement [18]. Therefore, more studies are further warranted to confirm the dose and frequency of MSCs administration to control glucose levels. Another important factor in this investigation scenario is the engineered-MSCs, aiming to boost cell reparative efficiency in association with more resilience in HG and oxidative stress microenvironment.

Classically, DKD causes several time-dependent histological alterations in kidney tissue. The mesangial expansion, one of the DKD pathophysiology hallmarks, was increased in BTBR*^ob/ob^* mice, indicated by periodic acid-Schiff (PAS) accumulation in the mesangium, whereas MSC-treated BTBR*^ob/ob^* mice had a decrease in PAS staining. MSCs are effective at reducing mesangial expansion through secretome factors, mainly through induction of VEGF expression, in diabetic pre-clinical models [26]. GMCs are responsible for the extracellular mesangial matrix synthesis and suffer cell cycle progression arrestment under hyperglycemic stress, eliciting extracellular matrix overproduction and cell exhaustion, which contributes to mesangial expansion [41]. Furthermore, related to glomeruli structure and function in DKD, we evaluated podocytes maintenance in BTBR*^ob/ob^* mice. As expected, there was a podocyte/glomerulus ratio decrease in consequence of DKD in BTBR*^ob/ob^* mice. MSC-treated mice exhibited increased podocyte/glomerulus in comparison to not treated animals. Podocytes preservation is an important and potential reason underlying the curtailed albuminuria observed in MSC-treated mice that could be obtained through cell death inhibition processes [42]. Podocytes could also be protected from HG damage and death induction by mitochondrial homeostasis recovery through biogenesis and mitophagy promotion [43].

In relation to mitochondrial molecular pathways, we evidenced alterations in the expression of MQCP related-genes by stress-media conditioning and DKD pathophysiological alterations. Our results document an inhibitory impact of HG, NP, and HP media on biogenesis and mitophagy-related genes expression. Despite the limitation that we have not investigated protein expression of these MQCP molecular targets, MSCs were reported to induce and recover the mitigated mitophagy PINK1 protein expression in cells conditioned to HG media [44]. Furthermore, the same promoting effect was observed in MSC-treated endothelial cells, which had recovered PGC-1α protein expression from previous HG-mediated downregulation [45]. Those evidences indicate that modulation of MQCP gene expression can be directly associated with protein expression changes as well, following MSC therapy. In a stress and damage scenario, both mitochondrial processes are essential for the proper adaptation aimed at cell reestablishment. Disturbances in the tightly regulated process of mitophagy and biogenesis balance aggravate cell injury facing hyperglycemia and oxidative stress [46]. The investigation of anti-oxidative therapy is one of the most applied strategies to overcome redox imbalance, but the literature is filled with controversies about this approach due to the excessive and broad anti-oxidative action, which culminates in the reductive stress [47]. Reestablishment of redox balance implies preservation of the physiological and naturally produced ROS, intrinsically involved in cell signaling processes, instead of oxidative-agents ablation [48]. MSC therapy has the great potential to restitute redox state through mitochondrial modulation and anti-oxidative effects [49]. Our results indicated that MSCs induced the recovery of mitophagy-related genes PINK1 and PARKIN expressions, in addition to inducing the expression of TFAM, a gene related to the biogenesis process, which has important biological implications. Mitophagy activation is a pro-survival mechanism that removes dysfunctional mitochondria debris that can, in association with the sustained formation of novel mitochondrial structure through biogenesis, renew mitochondrial networks [50].

In relation to BTBR*^ob/ob^* mice kidney cortexes, the expression of MQCP related-genes had an initial compensatory upregulation effect at 10 week-old, yet decreasing in a time-dependent manner until the 20 week-age. This effect was detected in all genes evaluated. Thus, MSC-treated mice had a marked gene up-regulation of PARKIN and PINK (mitophagy); MFN1 and OPA1 (outer and inner mitochondrial membrane fusion, respectively); DRP1 (mitochondrial membrane fission); KINESIN and DYNEIN (intracellular mitochondrial motility), and MIRO1 (mitochondrial transfer to neighbor cells). Therefore, that finding could be explained by mitochondrial matrix remodeling induction orchestrated by MSCs’ secretome and mitochondrial effects. MSCs have been shown to promote MQCP-related genes, inducing mitophagy [44] and biogenesis [15] in the diabetic setting. Moreover, MSCs can prevent mitochondria fragmentation, which represents one of the described consequences of HG and oxidative stress milieu impact [51], and ultimately inducing the fusion process [49]. Conversely, MSCs have demonstrated effects on fission stimulation through DRP1 gene expression [52], which is equally important for mitophagy and cell hemostasis. Of importance, these MQCP processes should be analyzed in a coordinated fashion, as they are interdependent.

Additionally, in relation to kidney tissue apoptosis and autophagy process, our results documented a potential suppressing effect of MSC treatment on the over activation of kidney cell death, as reduced cleaved caspase-3 protein expression, which were over activated in the later DKD stage of 20 week-old BTBR*^ob/ob^* mice. Of note, the induced caspase-3 protein in BTBR wild type mice was not followed by actual cell death pathway activation through caspase-3 cleavage, demonstrating a basal maintenance of this pathway signaling, though only activated when there was substantial DKD-driven stress and damage. There are compelling evidences demonstrating MSCs as an efficient intervention to diminish cell death in kidney tissue facing DKD, culminating in podocyte maintenance [42], and also downregulating the over induction of the autophagy pathway [53]. In addition to those results, we showed a suppressed LC3 overexpression in MSC-treated BTBR*^ob/ob^* mice at 14–15 weeks old. These attainable curtailing effects of MSCs on tissue over induced recycling and removal of damaged sections can result in decreasing structural damage and in amelioration of functional parameters, while fostering biogenesis pathways, as mitochondria renewal [30]. Autophagy is an intracellular degradation system that maintains intracellular homeostasis by removing damaged proteins and organelles, yet that biological process has a double-edged sword effect in the DKD setting. Therefore, autophagy downregulation has been clinically evidenced during the first stages of DKD due to HG-driven mTORC1 (mammalian target of rapamycin complex 1) induction in podocytes [54]. Obesity itself is also reported as a suppressor of autophagy through mTORC1 activation in proximal tubules [55]. Mechanistically, hyperactivation of mTORC1 induces endoplasmic reticulum stress and epithelial-mesenchymal transition phenotype in podocytes, mesangial expansion, and glomerular membrane basal thickening, so that rapamycin treatment or podocyte-specific knockout for Raptor, an essential component of mTORC1, protects the progression of early stages of DKD [54,56]. Therefore, improper mTORC1 activation in podocytes under diabetic conditions is crucial for podocyte injury and the genesis of proteinuria and may also cause dysfunction of other glomerular cells and tubular cells. Nonetheless, chronic inhibition of mTORC1 promotes imbalance of the mTOR signaling pathway and may aggravate glomerular and tubular lesions [54]. Additionally, persistent oxidative stress can eventually trigger a compensatory effect of kidney tissue in response to excessive ROS signaling, stimulating autophagy independently of mTORC1 [57]. The highly expressed autophagy in later stages, as we have observed in 20 week-old BTBR*^ob/ob^* mice, may lead to tissue loss of function and contribute to disease pathology. Therefore, autophagy has a complex relation with disease physiopathology and timing along disease progression, making it challenging to assess whether it should be up- or downregulated to promote repair [58].

Regarding the HG-driven persistent oxidative stress, MSC failed to reduce lipid peroxidation (4-HNE) in BTBR*^ob/ob^* mice. As a consequence of DKD progression, BTBR*^ob/ob^* mice have accumulation of ROS in kidney tissue as superoxide radicals and increased lipid peroxidation [59]. HG is associated with increased oxidative stress caused by over-production of NADH and ROS, which led to inhibition of glucose metabolism via glycolysis and tricarboxylic cycle [60]. Consequently, alternative glucose metabolic pathways, including polyol and hexosamine biosynthetic pathways, are activated and increase ROS production, thus completing the vicious circle of cellular oxidative stress. There is an increasing body of evidence showing the potential of MSCs as an effective anti-oxidative therapeutic tool against DKD progression [61,62]. However, our diabetic and obese mice exhibited severe hyperglycemia throughout the study, which could have abrogated MSC therapeutic potential in reducing oxidative stress. Importantly, MSC efficiency may be affected by other factors, such as the number of infusions, route of delivery, homing capacity, microenvironment, and the severity of the condition [62]. Therefore, MSCs systemically (intravenous) administrated have been clinically effective in reducing insulin intake of T2DM patients, with limited duration though [63], which could be explained by host factors, such as chronic hyperglycemia, sustained oxidative stress and inflammation, and by MSC properties, including being trapped in the lungs, lower levels of proliferation and migration, and higher rates of apoptosis and senescence in DM setting [62]. We performed MSC therapy without glycemic control by medicaments, therefore challenging cells to endure and survive in a highly glycotoxic microenvironment. In accordance with these observations, MSCs themselves may have been affected by HG. Regarding the hampering effects of DM on MSCs, HG is reported to foster oxidative stress, apoptosis, senescence, autophagy dysregulation, and mitochondrial damage in these cells [60]. Therefore, sustained HG in BTBR*^ob/ob^* mice diminishes MSCs’ effects in the in vivo scenario, as our results showed. On the other hand, the obtained positive results are highlighted in consequence of the harsh conditions implied by the exacerbated HG environment, indicating the therapeutic potential of MSCs against DM and DKD progression. In that way, further studies with MSC administration associated with glycemic control in the BTBR*^ob/ob^* mice will significantly improve the metabolic and renal therapeutic effects, halting DKD progression and more precisely simulating clinical translational application. Furthermore, it will be a great opportunity to investigate MSCs’ reparative mechanisms in a glucose-controlled setting, which can protect administered cells and enhance their therapeutic effectiveness [64].

## 4. Materials and Methods

### 4.1. Immortalized Glomerular Mesangial Cells (GMCs)

Mouse immortalized GMCs (SV40 MES 13, CRL-1927—ATCC, Gaitherburg, MD, USA), were cultured in Dulbecco’s Modified Eagle Medium (DMEM/F12) (Invitrogen, Carlsbad, CA, USA) supplemented with 5% fetal bovine serum (FBS, #F063—Cultilab, Campinas, SP, Brazil) and 1% penicillin/streptomycin (PS; #15140122—Gibco, Thermo Fisher Scientific, San Diego, CA, USA). Cells were split when they reached ~90% confluence. The medium was changed every other day. All cells were cultured at 37 °C in 98% humidified air containing 5% CO_2_.

For experimental conditioning, GMCs were cultured for 6 h and 72 h in DMEM 0.5% FBS (#F063—Cultilab, Campinas, SP, Brazil) supplemented with: (a) 1 g/L glucose (normal glucose—NG); (b) 5.4 g/L glucose (hyper glucose—HG); (c) 1 g/L glucose and 5.46 g/L mannitol (osmolarity control—MN); (d) 1 g/L glucose and 1 µL/mL H_2_O_2_ 0.03% *v/v* (normal glucose + hydrogen peroxide—NP); (e) 5.4 g/L glucose and 1 µL/mL H_2_O_2_ 0.03% *v/v* (hyper glucose + hydrogen peroxide—HP). Culture media were changed after 48 h.

### 4.2. Mesenchymal Stem Cells (MSCs)

MSCs were obtained from male 6–8 week old mT/mG mice (#007576; JAX Laboratories, Bar Harbor, ME, USA). These cells were extracted from femoral/tibial bone marrow (BM) flush, followed by purification in culture through passages in DMEM/F12 1:1 Ham (Invitrogen, Carlsbad, CA, USA) supplemented with 20% FBS (#F063—Cultilab, Campinas, SP, Brazil) and 1% PS (#15140122—Gibco, Thermo Fisher Scientific, San Diego, CA USA). BM-MSCs were characterized by fibroblast-like morphology and adherence to plastic in standard culture conditions, flow cytometry (Attune NxT Flow Cytometer—Thermo Fisher Scientific) immunophenotyping (CD29—# 11-0291-82; CD31—#11-0311-82; CD34—#11-0341-82; CD44—#11-0441-82; CD45—#11-0451-82; CD90—#11-0902-82; CD105—# MA1-80943; SCA-1—#11-5981-8; eBioscience, Thermo Fisher Scientific, San Diego, CA, USA) and multidifferentiation assay (chondrogenic, adipogenic and osteogenic lineages), as documented elsewhere [60,65,66,67]. MSCs are characterized by the expression of the surface molecules CD29, CD44, C90, CD105, and SCA-1 in the absence of CD34 and C45 (hematopoietic stem cell markers) and CD31 (endothelial cells marker).

After GMC conditioning in experimental media (NG, MN, HG, NP, and HP), MSCs were added to cell plates and both cell types were directly co-cultured for 24 h, following 1 MSCs:5 GMCs proportion, in DMEM/F12 medium (Invitrogen, Carlsbad, CA, USA) supplemented with 0.5% FBS (#F063—Cultilab, Campinas, SP, Brazil) and 1% PS (#15140122—Gibco, Thermo Fisher Scientific, San Diego, CA USA).

For conditioning experiments, we used 0.5% FBS in order to avoid GMC overgrowth and cell layer folding up. Therefore, these cells did not come off the plate.

### 4.3. BTBR^ob/ob^ Mice

Male mice BTBR.Cg-Lep^ob^/WiscJ (referred as BTBR*^ob/ob^*; #004824-JAX Laboratories, Bar Harbor, ME, USA), homozygous for leptin gene knockout. BTBR*^ob/ob^* is a reversible model for T2DM and DKD, characterized by hyperphagy, early development of obesity, insulin resistance, and hyperglycemia (6th week of age), followed by DKD establishment with time-dependent proteinuria (8th week of age), resembling human DKD alterations [17]. We evaluated BTBR WT mice as the healthy control, comparing them with BTBR*^ob/ob^* at different ages (10–11, 14–15, and 18–20 weeks old). Mice were housed in collective cages, with free access to regular chow (Nuvilab CR-1 irradiated, Quimtia S/A, Brazil) and tap water ad libitum, maintained in a temperature-controlled environment (23 °C) on a 12-h light/dark cycle. All experiments were performed in accordance with relevant guidelines and regulations defined by AAALAC. The respective Institutional Animal Care and Use Committees of Hospital Israelita Albert Einstein approved all procedures involving animals (15 December 2017) and the study was registered on the Jewish Institute of Research and Education, Hospital Israelita Albert Einstein, São Paulo, SP, Brazil (No. 3125-17).

### 4.4. MSCs Administration

Rapidly thawed MSC vials were evaluated in terms of cell viability by trypan blue staining (accepted if >90%). BTBR*^ob/ob^* mice received BM-MSC injections, containing 1 × 10^6^ cells/each in warm saline, at the 8th and 10th weeks of age, through intraperitoneal route. We evaluated MSC therapy effects on BTBR*^ob/ob^* mice after 4 weeks (14–15 weeks old) and after 8–10 weeks (18–20 week-old mice) of cell injections.

### 4.5. Cell Death

Cell death induction in GMCs was evaluated by Annexin-V (FITC^+^; #V13241—Thermo Fisher Scientific, Waltham, MA, USA), propidium iodide (PE^+^; #V13241—Thermo Fisher Scientific, Waltham, MA, USA) and DAPI (Violet^+^; #62248—Thermo Fisher Scientific, Waltham, MA, USA) staining by flow cytometry (Attune NxT cytometer, Invitrogen; FlowJo software, BD Biosciences). All experiments were done three times in triplicate and the mean of each experiment was calculated.

### 4.6. Mitochondrial-H_2_O_2_

For quantification (%) of GMCs with H_2_O_2_ accumulated specifically in mitochondria we used MitoPY1 (FITC^+^; #SML0734—Sigma-Aldrich, St. Louis, MO, USA) by flow cytometry (Attune NxT cytometer, Invitrogen; FlowJo software, BD Biosciences). All experiments were done three times in triplicate and the mean of each experiment was calculated.

### 4.7. Mitochondrial Membrane Polarization

Polarized/depolarized mitochondria ratio in GMCs was evaluated by MitoProbe JC1 (#M34152—Thermo Fisher Scientific, Waltham, MA, USA) by flow cytometry (Attune NxT cytometer, Invitrogen and FlowJo software, BD Biosciences). Functional (polarized) mitochondria attract and aggregate JC1 probe, which shifts fluorescence emission from 530 nm (diffused probe—FITC^+^) to 590 nm (oligomerized probe—PE^+^). Results were expressed as PE^+^/FITC^+^ ratio. All experiments were done three times in triplicate and the mean of each experiment was calculated.

### 4.8. Mitochondrial Quality Control Program (MQCP) Targets

RNA was extracted from BTBR*^ob/ob^* mice kidney cortexes and GMCs (RNeasy mini kit; #74106—QIAGEN, Hilden, Germany), quantified (10 ng/µL; NanoDrop, Thermo Fisher Scientific) and used to synthesize cDNA (High-Capacity cDNA Reverse Transcription Kit; #4368814—Applied Biosystems, Waltham, MA, USA).

qPCR reaction (TaqMan Gene Expression Master Mix; #4369542—Applied Biosystems, Waltham, MO, USA) was performed using QuantStudio 6 Flex Real-Time PCR System (Applied Biosystems). GUSB was selected as the endogenous housekeeping gene (#Mm01197698_m1, Taqman Endogenous Control—Applied Biosystems, Waltham, MA, USA) and fold change was calculated as relative gene expression of mitochondrial quality control program targets: PGC1α and TFAM for biogenesis; PARKIN and PINK1 for mitophagy; DRP1 and FIS1 for fission; MFN1/2 and OPA1 for fusion; and MIRO1, MIRO2, DYNEIN and KINESIN for motility analysis. Absolute number of transcripts was calculated by 2^ΔΔCt^ method. Genes of BTBR*^ob/ob^* mice were normalized to BTBR WT mice.

### 4.9. Metabolic and Renal Functional Analysis

Blood collection was performed at baseline, week 4 and week 8 post-MSC injections for fasting glucose measurement (Accu-Chek, Performa, São Paulo, SP, Brazil). Urine was collected at baseline, week 4 and week 8 post-MSC injections using a metabolic cage; albuminuria was measured by ELISA (Mouse Albumin ELISA Kit; #ab207620—Abcam, Cambridge, MA, USA). Albumin levels were normalized to urine creatinine, quantified by biochemistry (#1010—Creatinine K Vet, Labtest, Lagoa Santa, MG, Brazil) using Cobas Mira Plus (Roche). Results were expressed as urine albumin-to-creatinine ratio (UACR; g/mg). BTBR*^ob/ob^* mice were weighed twice every week.

### 4.10. Mesangial Expansion

BTBR*^ob/ob^* and wild type kidney sections (paraffin-fixed, 3–4 µm thick) were stained with periodic acid-Schiff (PAS) trichrome staining in each experimental group. Sections were then analyzed by light microscopy (magnification, 40×). A quantitative analysis of mesangial expansion was performed. The increase in mesangial matrix was determined by the presence of PAS-positive area in the mesangium, and was expressed as a percentage. The glomerular area (μm^2^) was also traced along the outline of capillary loops using CellSens software (Olympus) in 30 randomly selected glomeruli in each animal.

### 4.11. Immunohistochemistry (IHC) Analysis

BTBR*^ob/ob^* and wild type mice kidneys were fixed with formalin (10%) and sectioned at 3–4 µm thick. IHC reaction was performed with EnVision FLEX High pH kit (#K8000—DAKO, Carpinteria, CA, USA). Scoring for WT-1^+^ cells was carried out by counting the number of positive nuclei in 25 glomeruli randomly chosen in kidney cortexes and outer medulla sections using 10× magnification and after applying a rabbit polyclonal WT-1 antibody (anti-WT1; #sc-192—Santa Cruz, Dallas, TX, USA). Data from all fields and all kidneys were pooled to obtain the number of WT-1^+^ podocytes per glomerular cross-section. For autophagy induction, quantification of LC3 (anti-LC3; #4445S—Cell Signaling, Danvers, MA, USA) was performed analyzing cortex and medulla regions. For apoptosis pathway analysis, we evaluated caspase-3 and cleaved caspase-3 (#9662; #9661—Cell Signaling, Danvers, MA, USA) protein activation. Oxidative stress was measured by 4-HNE staining (anti-4-hydroxynonenal polyclonal antibody; #ab46545—Abcam, Cambridge, MA, USA) in kidney sections. The intensity of staining was determined using the optical density function of CellSens software (Olympus). Tissue sections were counter-stained with Mayer’s hemalum.

### 4.12. Statistical Analysis

Results are expressed as mean ± SEM. Statistical analysis was performed following normality test by Shapiro–Wilk. In case of normal distribution, ANOVA one-way was performed, followed by post-test of Brown Forsythe and Welch. Two-way ANOVA was applied to repeated measures analysis, followed by Holm–Sidak post-test for multiple comparisons; or Fisher (least significant difference) LSD post-test, in order to obtain statistical power when facing sample variability. In case of non-normal distribution, we applied Kruskall–Wallis or multiple t-test with Holm–Sidak post-test for multiple comparisons. For qRT PCR analysis of MQCP-genes expression in GMC samples, we performed two-way ANOVA analysis or Kruskall–Wallis test with Holm–Sidak post-test, using *n* = 2 per group. All tests were performed using GraphPad Prism (GraphPad Software, San Diego, CA, USA). A *p*-value < 0.05 was considered statistically significant.

## 5. Conclusions

Here, our study documented the progressive deleterious effects of HG and the related oxidative stress in DKD in both pre-clinical and in vitro models. We have efficiently applied MSC therapy with safety and efficacy for suppressing DKD-induced damage. Our findings have important therapeutic implications, providing insights into cellular and molecular mechanisms of MSC-based therapy in a DKD setting.
